# Effects of an intensive pulmonary rehabilitation program on postoperative pulmonary complications in frail elderly patients undergoing hepatobiliary and pancreatic surgery: a randomized controlled trial

**DOI:** 10.3389/fonc.2026.1791031

**Published:** 2026-08-17

**Authors:** Jinhua Feng, Fuyu Li, Huan Feng, Hui Ye, Min Gao, Yuanchun Ling, Haijie Hu, Qiang Han, Bairong Shen, Ka Li

**Affiliations:** 1 Department of Biliary Surgery, West China School of Medicine, West China Hospital, Sichuan University, Chengdu, China; 2 West China School of Nursing, Sichuan University, Chengdu, China; 3 Institutes for Systems Genetics, Frontiers Science Center for Disease-related Molecular Network, West China Hospital, Sichuan University, Chengdu, China

**Keywords:** compliance, hepatobiliary and pancreatic surgery, intensive pulmonary rehabilitation exercises, postoperative outcome, postoperative pulmonary complications

## Abstract

**Background:**

Frail elderly patients undergoing hepatobiliary-pancreatic (HBP) surgery face a substantially elevated risk of postoperative pulmonary complications (PPCs), which are associated with increased mortality, extended morbidity, and greater healthcare costs. Although the “I Cough” pulmonary rehabilitation program is a well-established preventive strategy for PPCs, its clinical effectiveness is often limited by suboptimal patient adherence. This randomized controlled trial was therefore conducted to determine whether a nurse-led intensive pulmonary rehabilitation exercises (IPREs) program could improve compliance and reduce the occurrence of PPCs in this highly susceptible population.

**Methods:**

A total of 160 participants were randomly assigned to two groups. Eighty patients in the intervention (IPREs) group received IPREs, whereas 80 patients in the control group underwent conventional self-pulmonary rehabilitation training. The incidences of PPCs and other postoperative outcomes were compared between the two groups.

**Results:**

The median compliance rate with pulmonary rehabilitation exercises was significantly higher in the IPREs group than in the control group (100.00% [IQR 83.33–100.00%] vs. 33.33% [IQR 33.33–66.67%]; *P* < 0.001). The incidence of PPCs was significantly lower in the IPREs group (22.50% vs. 38.75%; OR 0.459, 95% CI 0.230–0.926; P = 0.026), with notably reduced rates of pneumonia and atelectasis (*P* < 0.05). The overall rate of major complications was also reduced in the IPREs group (12.50% vs. 25.00%; OR 0.429, 95% CI 0.186–0.989; P = 0.043). Additionally, the median postoperative hospital stay was shorter in the IPREs group (6 days [IQR 5–8] vs. 8 days [IQR 6–9]; *P* = 0.035).

**Conclusions:**

The nurse-led intensive pulmonary rehabilitation program significantly improved compliance with pulmonary rehabilitation exercises, reduced the incidence of PPCs, and shortened postoperative hospitalization in frail elderly patients undergoing HBP surgery.

**Clinical trial registration:**

https://www.chictr.org.cn, identifier ChiCTR2000040021.

## 1 Introduction

Hepatobiliary and pancreatic (HBP) malignancies are prevalent among elderly individuals (1, 2). Surgical resection remains the primary curative treatment (3), but these complex procedures involve substantial tissue trauma and prolonged operative duration, leading to a high risk of postoperative complications. Among these, postoperative pulmonary complications (PPCs) are the most frequent adverse events after such major abdominal surgery, affecting up to 30% of patients (4, 5). These complications significantly impair recovery outcomes and escalate healthcare resource utilization and economic costs (6). Frail elderly patients with nutritional deficits, reduced cardiopulmonary functional reserve, and multiple comorbidities are particularly vulnerable to PPCs following HBP surgery (7, 8), which are strongly associated with increased mortality, morbidity, and healthcare costs (9, 10). Therefore, effective prevention of PPCs in this high-risk population represents a critical objective in perioperative management.

Perioperative respiratory exercises are evidence-based nursing interventions proven to reduce PPCs (11). The “I Cough” program is a classic standardized perioperative pulmonary care protocol. Its acronym represents the key components of the intervention: Incentive spirometry (IS), Coughing and deep breathing, Oral care, Understanding (patient, family, and staff education), Getting up early (mobilization), and Head-of-bed elevation (12). Most studies indicate that this program effectively reduces the incidence of postoperative pneumonia and unplanned intubation, while shortening hospital stays (13, 14). However, the clinical efficacy of the program over the long term has been limited by variable patient adherence to self-managed rehabilitation exercises. Current reports highlight substantial heterogeneity in the implementation of “I Cough” across clinical settings, particularly regarding exercise modalities, methods, frequency, and duration (14, 15). An audit of “I Cough” nursing practices revealed that only 29% of patients complied with the standard protocol, and merely 20% were out of bed (sitting or walking) during survey visits (12). Evidence suggests that adherence to “I Cough” principles is inversely correlated with the incidence of PPCs (15). Frail elderly patients frequently exhibit diminished physiological reserve. The trauma and stress associated with HBP surgery can further exacerbate their frailty, posing significant challenges to effective implementation of pulmonary exercise protocols (12). To date, ensuring sustained adherence to the “I Cough” protocol among this vulnerable population has received inadequate attention. With the growing adoption of prehabilitation and chronic disease management programs, nurses have expanded their roles to serve as consultants, educators, and therapists (11, 16). Nurse-led rehabilitation training has demonstrated favorable clinical outcomes and cost-effectiveness (16, 17). However, whether a nurse-led, intensive pulmonary rehabilitation exercises (IPREs) program can reduce the incidence of PPCs in frail elderly patients undergoing HBP malignancy surgery remains uncertain.

This study focuses on frail elderly patients with HBP malignancies and aims to evaluate the efficacy of a nurse-led, intensive “I Cough” program in reducing the incidence of PPCs. The findings are expected to provide an evidence-based strategy for improving postoperative recovery in this high-risk population.

## 2 Materials and methods

### 2.1 Trial design

This study was a two-arm randomized controlled trial (RCT). The study protocol was approved by the Ethics Committee of West China Hospital of Sichuan University with ethical approval number 2019/1169. The study complied with the Declaration of Helsinki and was registered at (identity number ChiCTR2000040021). Written informed consent was obtained from all eligible participants before randomization, and the study was performed in accordance with the guidelines for parallel RCTs (18).

### 2.2 Settings and participants

This study was conducted in the Department of Biliary Surgery at West China Hospital, Sichuan University, from June 2023 to October 2025. A total of 160 hospitalized elderly patients with HBP cancers who met the eligibility criteria were enrolled. The inclusion criteria were as follows: age ≥65 years; a FRAIL score of 3 or higher (indicating frailty); and a preoperative diagnosis of liver, biliary tract, or pancreatic cancer; scheduled for elective HBP surgery; no prior radiotherapy or chemotherapy; no liver or kidney failure or cardiopulmonary insufficiency; American Society of Anesthesiologists (ASA) grade I–III; and no evidence of distant metastasis on abdominal CT. In addition, patients who were unable to ambulate for more than 10 minutes preoperatively were also excluded.

### 2.3 Sample size

The primary outcome was the incidence of PPCs, compared between the intervention and control groups. Sample size estimation was based on detecting a clinically meaningful difference in PPC rates using two-sample noninferiority or superiority tests, following the approach described by Park et al. (4). An effect size enabling 80% power at a significance level of ***P ≤*** 0.05 was used to determine the optimal sample size. Pocock’s formula, designed for comparing proportions (P_1_ and P_2_) in two equally sized groups, yielded a requirement of 73 patients per group. To enhance the robustness of the trial, the sample size was increased to 80 patients per group, consistent with methodologies employed in prior studies.

### 2.4 Randomization and blinding

An independent statistician generated the computer-based randomization sequence. Eligible participants were randomly allocated in a 1:1 ratio to either the IPREs group or the control group. A study coordinator prepared sequentially numbered, opaque, sealed envelopes containing the allocation assignments, which were opened only after written informed consent was obtained. To minimize bias, personnel involved in group allocation, data collection, and statistical analysis were blinded to the group assignments. Furthermore, participants from the two groups were accommodated in separate hospital rooms to prevent cross-contamination. However, due to the nature of the intervention, neither the patients nor the nursing staff administering the pulmonary rehabilitation could be blinded, resulting in a single-blind study design.

### 2.5 Standard care

Except the perioperative pulmonary rehabilitation training intervention, the other management programs used to treat the two groups were the same. The patients were treated and received nursing care from the same medical team during hospitalization. The same team of surgeons performed surgery combined with general anesthesia. All patients received protective ventilation, along with a standardized enhanced recovery after surgery (ERAS) protocol applied preoperatively, intraoperatively, and postoperatively (19, 20).

### 2.6 Intervention

Upon admission, all participants underwent a standardized assessment for PPCs using the Assess Respiratory Risk in Surgical Patients in Catalonia (ARISCAT) risk scale. Subsequently, participants in both groups received a same set of materials, including an incentive spirometer (IS), a grip strength trainer, and an information booklet. All participants then attended a 30-minute coaching session delivered by a rehabilitation nurse, covering the components detailed in **Table 1**.

**Table 1:** Overview of the standardized 30-minute coaching session following hospital admission.

➢ Education about the possibility of PPCs after surgery. ➢ Coaching on pulmonary rehabilitation training according to the information booklet. ➢ Instruction on how to use the IS and grip trainer. Every participant mastered the technique, as judged by the rehabilitation nurse, who set the individual IS inspiratory volume training and grip strength training goals and encouraged the participants to achieve their goals in every training session. Tutoring on the performance of prescribed breathing exercises and activity training was provided, along with demonstrations of self-directed breathing and activity exercises as outlined in the accompanying booklet.

PPCs, postoperative pulmonary complications; IS, incentive spirometer.

The information booklet provided detailed written and visual instructions on the “I Cough” program. Participants randomized to the IPREs group received additional support from a dedicated rehabilitation nurse, who delivered face-to-face interventions at least twice daily over a 5-day period (from one day preoperatively to four days postoperatively). In contrast, patients in the control group performed self-managed pulmonary rehabilitation exercises. A comprehensive overview of the interventions administered in both groups is presented in **Table 2**.

**Table 2:** Comparison of perioperative “I Cough” protocols implementation between the IPREs group and the control group.

“I Cough” protocols	IPREs group	Control group
1. Incentive spirometry Exercise IS as close to the target value as possible, 10 times every hour (3–5 efforts per set) while awake	➢ The rehabilitation nurse coached each participant on IS training once every morning and once every afternoon. ➢ Document the IS inspiratory volume every 4 h while awake.	➢ Patient self-training according to the information booklet. ➢ Document the IS inspiratory volume every 4 h while awake.
2 Coughing and deep breathing (Each set of 10 breaths was followed by three coughs. Patients were encouraged to cough and breathe deeply every 2 h)	➢The rehabilitation nurse provided face-to-face instruction at least twice a day.	➢ Patient self-training according to the information booklet.
3. Oral care Brushing teeth and using mouth wash twice daily	➢ The rehabilitation nurse provided instruction twice daily (usually 8 AM and 8 PM).	➢ Patient self-management.
4. Understanding Patient and caregiver education	➢ The rehabilitation nurse led 10-minute coaching sessions every day, which included the following: a. Education about the possibility of PPCs after surgery; b. Coaching on pulmonary rehabilitation training according to the information booklet; c. Feedback on pulmonary rehabilitation training.	➢ The charge nurse conducted daily routine education on preventing complications.
5. Getting out of bed and activity Preoperative walking training >1 hour. Grip strength training for 10 minutes/hour when awake. Early postoperative activity, progress activity to ambulate at least 3 times/d.	➢ Grip strength training for 10 minutes/hour when awake. The rehabilitation nurse provided face-to-face instruction at least two times a day. ➢ The rehabilitation nurse accompanied the patient while walking ≥ 20 min or ≥ 2000 m at a pace causing mild breathlessness, once every morning and afternoon. ➢The rehabilitation nurse provided face-to-face instruction regarding mobilization and early activities after surgery: a. Training grip strength for 10 minutes/hour when awake from POD 1; b. Early walking was encouraged in the first 24 h postoperatively (getting out of bed, going to the bathroom, walking along the corridor if the patient was alert and it was safe to do so); c. Transitioning from the bed to a chair at least 3 times/d, preferably at mealtimes, with assistance as needed, and progress activity to ambulation at least 3 times/d, with assistance as needed; d. Outside these assisted sessions, participants were advised to walk or exercise by their bedside as frequently as they were able.	➢ The charge nurse encouraged patients to get out of bed and engage in activities. ➢Caregiver or patient self-management according to the information booklet.
6. Head-of-bed elevation (>30°)	➢ The rehabilitation nurse checked bed head height	➢ Caregiver or patient self-management
Others: Pain control	Routine analgesia protocol: During POD 0-3, PCA with NSAIDs every 12 hours. During POD 4-5: NSAIDs i.v. as needed, max 2 doses/day. Stepwise rescue protocol:VAS >3: tramadol (100 mg) i.m.VAS >5: dezocine (5 mg) i.m. or another opioid. Note: The NSAIDs administered were selective COX-2 inhibitors. Their administration was contraindicated in patients with renal impairment (eGFR < 60 mL/min/1.73 m²), a history of peptic ulcer disease, or significant coagulopathy. All patients received daily serum creatinine monitoring and bleeding assessments. For patients with contraindications, weak opioids were prioritized for analgesia based on clinical evaluation.	

IPREs, intensive pulmonary rehabilitation exercises; IS, incentive spirometer; POD, postoperative day; PCA, patient-controlled analgesia; NSAIDs, nonsteroidal anti-inflammatory drugs; i.m., intramuscular; i.v., intravenous.

### 2.7 Measurements

The ARISCAT risk score scale was developed in 2010 using prospectively collected data from 59 Spanish hospitals (21). This scoring system incorporates seven pre- and intraoperative variables: age, preoperative SpO_2_, recent respiratory infection (within the past month), preoperative anemia, surgical approach (abdominal or thoracic), surgical duration, and surgery type (emergency or elective). The scoring system demonstrates strong predictive performance for PPCs. Patients are stratified into three risk categories according to the total score: low (≤25 points), intermediate (26–44 points), and high (>45 points) risk.

The FRAIL scale, created by Morley and colleagues, is a self-reported screening tool for assessing frailty (22). It consists of five components: fatigue, resistance, ambulation, illness, and weight loss. The total score ranges from 0 to 5, with 0 indicating nonfrailty, 1–2 indicating prefrailty, and 3 or higher representing frailty. This brief and easy to administer instrument is practical for identifying frailty in general practice and is recommended as a preferred frailty screening tool.

To accurately document patient adherence to the training program, each participant was provided with an activity training record book, which was completed through self-report and assessment by the Charge nurse. Compliance with each component of the “I Cough” protocol was evaluated as follows: if a component was performed according to the information booklet from one day preoperatively to four days post-operatively, it was marked as compliant; otherwise, it was considered noncompliant. Overall “I Cough” compliance was calculated as the percentage of compliant components out of the total. Full compliance (100%) was achieved only when all six major components were strictly followed.

Self-reported pain at rest and during mobilization was measured using a 10-cm horizontal visual analog scale (VAS) at four time points (Pre1, POD 1, POD 3, and POD 5). The scale had a vertical line at the left end for ‘no symptoms’ (score of 0) and at the right end for ‘worst possible experience’ (score of 10). When questioned about symptoms, patients marked their pain level on the line, and the distance from 0 to the mark represented the pain score. In addition to routine multimodal analgesia, all supplemental rescue analgesic doses were recorded.

### 2.8 Adverse events and safety monitoring

The chief surgeon monitored the anesthetic risks and perioperative complications of the participants, and the charge nurse immediately reported adverse events to the attending physician, who initiated the appropriate treatment.

### 2.9 Discharge criteria

Discharge timing was determined by the attending surgeon according to standardized criteria, which included stable vital signs, adequate analgesia with oral medications, independent ambulation, discontinuation of intravenous fluids, tolerance of a solid diet, spontaneous voiding, and return of bowel function.

### 2.10 Data collection and outcome definitions

#### 2.10.1 Primary outcome

The primary outcome was the incidence of PPCs, defined as the occurrence of any predefined complication within the first 14 days after surgery. PPCs included pneumonia, atelectasis, pulmonary embolism, pleural effusion, acute respiratory distress syndrome (ARDS), respiratory failure, and other related conditions. All participants were prospectively assessed daily for respiratory symptoms until postoperative day 14. Suspected cases underwent confirmatory diagnostic tests, such as CT scans or sputum cultures, when clinically indicated.

#### 2.10.2 Secondary outcomes

The secondary outcomes comprised compliance with the “I Cough” program, functional recovery indicators, other postoperative major complications (defined as Clavien–Dindo grade III or higher), and hospital utilization metrics in both groups. All patients were followed up for 30 days postoperatively. The records were thoroughly reviewed upon completion, and multiple follow-up attempts were made to contact participants with missing responses to minimize missing data.

### 2.11 Data analysis

All statistical analyses were conducted using SPSS (version 23.0; SPSS Inc., Chicago, IL, USA) in accordance with the intention-to-treat (ITT) principle. The normality of continuous variables was evaluated using the Shapiro–Wilk test. Normally distributed data are summarized as mean ± standard deviation (SD), and non-normally distributed data as median and interquartile range [M(IQR)]. Categorical variables are expressed as frequencies (percentages). Between-group comparisons were performed using Student’s t-test or the Mann–Whitney U test for continuous variables, depending on data distribution, and Pearson’s chi-square test, Wilcoxon rank-sum test, or Fisher’s exact test for categorical variables, as appropriate. For longitudinal analyses, a two way repeated measures ANOVA with time and group (IPREs vs. control) as factors was applied to normally distributed variables; non-normally distributed data were logarithmically transformed prior to analysis. *Post-hoc* comparisons at individual time points were conducted using the Kruskal–Wallis test. A two-sided *P*-value<0.05 was considered statistically significant.

## 3 Results

### 3.1 Baseline characteristics of the study population

**Figure 1:**
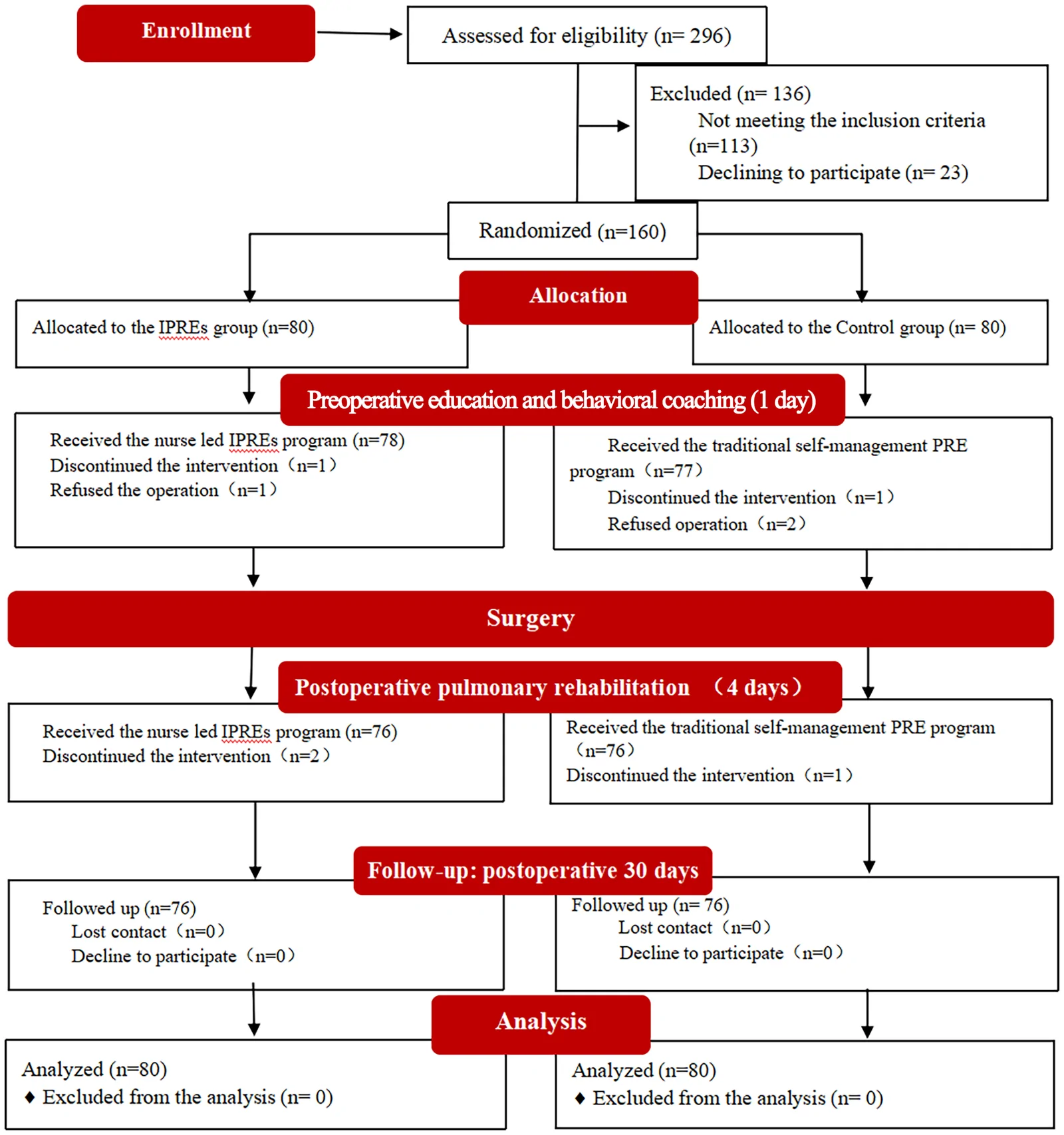
CONSORT flow chart of the study design. IPREs, intensive pulmonary rehabilitation exercises; PRE, pulmonary rehabilitation exercise.

A total of 296 patients were assessed for eligibility. Of these, 113 patients did not meet the inclusion criteria, and 23 declined to participate. Ultimately, 160 eligible participants were successfully recruited. Eighty participants were randomly assigned to the IPREs group, while the remaining participants were assigned to the control group. The CONSORT diagram of the study protocols is shown in **Figure 1**.

The study cohort comprised 108 men (67.50%) and 52 women (32.50%), with a mean age of 72.06 ± 5.23 years. Most tumors were classified as TNM stage I-II (73.75%), and the majority of patients (64.38%) had Child-Pugh class A liver function. The most prevalent comorbidities were hypertension (25.00%), diabetes (17.50%), and chronic obstructive pulmonary disease (16.25%). The distribution of surgical procedures was comparable between the two groups. Overall, no statistically significant differences were observed in baseline characteristics between the groups (*P*> 0.05), as detailed in **Table 3**.

**Table 3:** Comparison of baseline characteristics between the two groups.

Characteristics	Total (n=160)	IPREs group (n=80)	Control group (n=80)	*P*
Sex, n (%)				0.736
Male	108(67.50)	53 (66.25)	55 (68.75)	
Female	52(32.50)	27 (33.75)	25 (31.25)	
Age (years, mean ± SD)	72.06 ± 5.23	72.31 ± 5.65	71.11 ± 5.01	0.331
Body weight (kg, mean ± SD)	65.11 ± 8.45	63.21 ± 9.41	68.56 ± 10.78	0.621
BMI (kg/m2, M(IQR))	22.81(20.77, 24.26)	22.21(20.71, 23.66)	24.55 (21.21, 24.77)	0.310
ALB (g/L, M(IQR))	40.81(36.71–43.86)	41.12(36.77–44.12)	40.67 (37.21–43.43)	0.589
Hemoglobin (g/L, mean ± SD)	125.61 ± 22.16	123.75 ± 23.56	126.14 ± 21.33	0.761
Total bilirubin (μmol/L, M(IQR))	45.12(30.94–56.11)	41.20(31.34–50.10)	47.10 (26.93–46.88)	0.815
Aspartate aminotransferase (IU/L, mean ± SD)	40.19 ± 27.15	44.18 ± 22.15	37.56 ± 29.22	0.761
Comorbidity, n (%)				0.830
Hypertension	40(25.00)	22 (27.50)	18 (22.50)	
Diabetes	28(17.50)	12 (15.00)	16 (20.00)	
Coronary disease	13(8.13)	7(8.75)	6 (7.50)	
COPD	26(16.25)	15 (18.75)	11 (13.75)	
Other comorbidities	9(5.63)	4 (5.00)	5 (6.25)	
Child–Pugh liver function classification, n (%)				0.620
A	103(64.38)	53 (66.25)	50(6.25)	
B	57(35.63)	27 (33.75)	30(3.75)	
Disease				0.345
Liver cancer	65(40.63)	37 (46.25)	28 (35.00)	
Cholangiocarcinoma/Gallbladder cancer	61(38.13)	28 (35.00)	33 (41.25)	
Pancreatic cancer	34(21.25)	15 (18.75)	19 (23.75)	
TNM stage, n (%)				0.936
I	33(20.63)	16 (20.00)	17(21.25)	
II	83(51.88)	41 (51.25)	42 (52.50)	
III	44(27.50)	23 (28.75)	21 (26.25)	
Handgrip strength (kg, mean ± SD)	35.45 ± 11.31	34.21 ± 12.41	36.35 ± 10.18	0.612
Surgical procedure, n (%)				0.596
Hepatectomy	72(45.00)	39 (48.75)	33 (41.25)	
Biliary surgery	49(30.63)	22(27.50)	27(33.75)	
Pancreatectomy	39(24.38)	19(23.75)	20(25.00)	
Surgical approach, n (%)				
Open surgery	119(74.38)	59 (73.75)	60 (75.00)	0.856
Laparoscopic surgery	41(25.63)	21 (26.25)	20 (25.00)	
ASA classification, n (%)				
II	45(28.13)	21 (26.25)	24 (30.00)	0.845
III	83(51.88)	42 (52.50)	41 (51.25)	
IV	32(20.00)	17 (21.25)	15 (18.75)	
ARISCAT score				
Moderate risk (26–44 points)	93(58.13)	48 (60.00)	45 (56.25)	0.231
High risk (>45 points)	67(41.88)	32 (40.00)	35 (43.75)	
Anesthesia time (min)	272.65 ± 67.32	282.65 ± 64.15	266.78 ± 79.65	0.871
Blood loss (ml, M(IQR))	200 (100, 410)	200 (100, 410)	210 (100, 440)	0.657

IPREs, intensive pulmonary rehabilitation exercises; ALB, Albumin; BMI, body mass index; COPD, chronic obstructive pulmonary disease; TNM, tumor node metastasis; ASA, American society of anesthesiologists; ARISCAT, assess respiratory risk in surgical patients in catalonia; M(IQR), median (interquartile range).

### 3.2 Compliance with the “I Cough” program

Overall compliance with the “I Cough” program was 66.7% (IQR 33.3-83.3%). The IPREs group demonstrated significantly higher median compliance (100.0%; IQR 83.3-100.0%) compared with the control group (33.3%; IQR 33.3-66.7%; *P* < 0.001). Full protocol adherence was achieved by 54 patients (67.5%) in the IPREs group versus 10 patients (12.5%) in the control group. Component specific compliance was consistently superior in the IPREs group across all measures (*P* < 0.05). The control group exhibited particularly low adherence to mobilization, incentive spirometry, and coughing/deep breathing exercises, with rates ranging from approximately 20% to 27.5% (**Table 4**).

**Table 4:** Comparison of compliance with the “I Cough” protocol in the IPREs and control groups.

“I Cough” protocols	Overall (n=160)	IPREs group (*n* = 80)	Control group (n=80)	*P*
1. Incentive spirometry, n (%)	83 (51.88)	65 (81.25)	18 (22.50)	<0.001
2. Coughing and deep breathing, n (%)	84 (52.50)	62 (77.50)	22 (27.50)	<0.001
3. Oral care, n (%)	96 (60.00)	66 (82.50)	30 (37.50)	<0.001
4. Understanding, n (%)	137(85.63)	76 (95.00)	61 (76.25)	0.001
5. Getting out of bed and activity, n (%)	70 (43.75)	54 (67.50)	16 (20.00)	<0.001
6. Head-of-bed elevation, n (%)	137 (85.63)	74 (92.50)	65 (81.25)	0.035
Overall compliance with the “I Cough” protocol, M(IQR), % compliance	66.67 (33.33–83.33)	100.00 (83.33–100.00)	33.33 (33.33–66.67)	<0.001

Categorical data are described as numbers with percentages, and continuous data are described as medians and interquartile ranges; IPREs, intensive pulmonary rehabilitation exercises; M(IQR), median (interquartile range).

### 3.3 Functional recovery indicators

**Figure 2:**
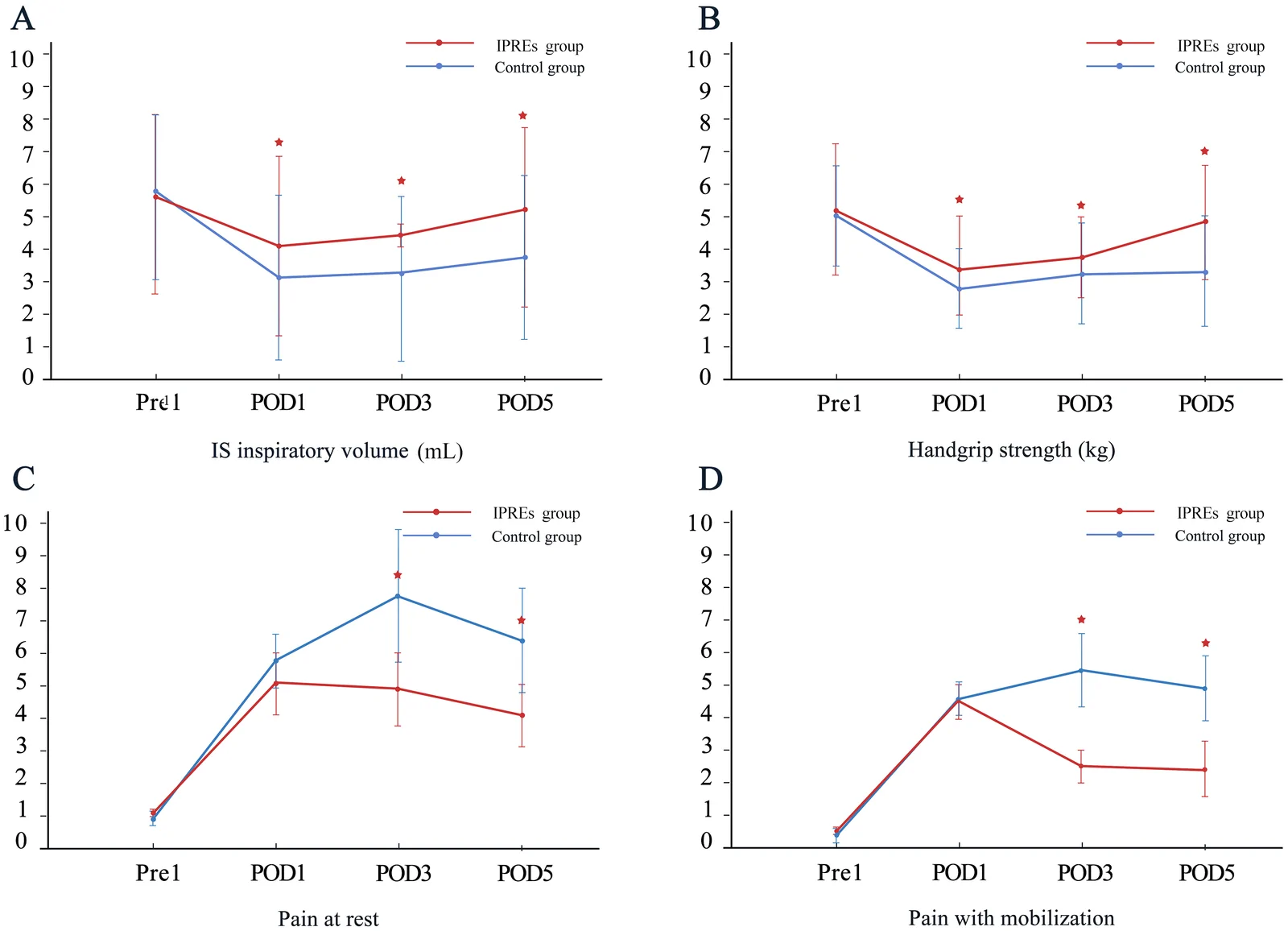
Temporal changes in functional recovery indicators between the two groups. **(A)** Inspiratory volume; **(B)** Grip strength; **(C)** Pain scores at rest; **(D)** Pain scores during mobilization. IPREs, intensive pulmonary rehabilitation exercises; IS, incentive spirometer; Pre, preoperative; POD, postoperative day. Repeated-measures ANOVA was used for the analysis. P values for the treatment difference between the IPREs group and the control group with respect to the time points were calculated when a significant treatment*time interaction was found. When a time*treatment interaction was not detected, the difference between treatments was tested by Tukey’s *post hoc* tests. * Data with different letters are significantly different between the IPREs group and the control group (*P* < 0.05).

Compared with the control group, patients receiving the IPREs intervention showed significantly higher IS inspiratory volumes and greater handgrip strength on postoperative days (POD) 1, 3, and 5 (all *P* < 0.05; **Figures 2A, B**). Pain scores at rest and during mobilization were also significantly lower in the IPREs group than in the control group on POD 3 and POD 5 (**Figures 2C, D**). The proportion of patients requiring additional analgesic doses was significantly higher in the control group (38.75% vs. 12.50%, P<0.01). Furthermore, patients in the IPREs group achieved independent ambulation earlier (42.31 ± 9.56 hours vs. 78.22 ± 10.21 hours, *P* < 0.001) and exhibited faster recovery of gastrointestinal function, as evidenced by a shorter time to first flatus (42.41 ± 8.11 hours vs. 78.14 ± 12.80 hours, *P* < 0.05) compared to the control group.

### 3.4 Postoperative pulmonary complications

**Figure 3:**
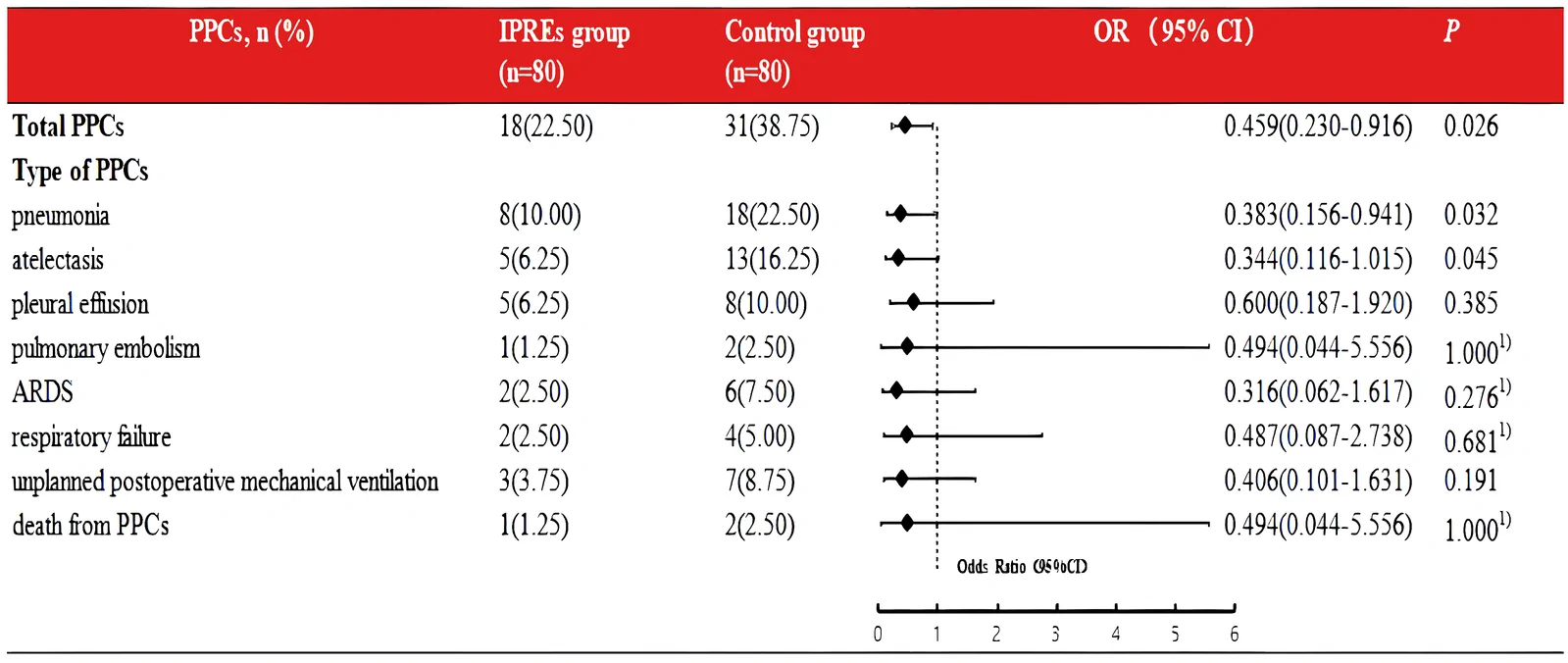
Comparison of postoperative pulmonary complications between the groups. IPREs, intensive pulmonary rehabilitation exercises; ARDS, acute respiratory distress syndrome; PPCs: postoperative pulmonary complications; POD: postoperative days; OR, odds ratio. ^1)^ Fisher’s exact probability.

PPCs occurred in 18 patients (22.50%) who received nurse-led pulmonary rehabilitation training and in 31 patients (38.75%) who received conventional self-management pulmonary rehabilitation training (odds ratio [OR], 0.459; 95% CI (0.230–0.8916); *P* = 0.026) (**Figure 3**). The most common PPCs were pneumonia and atelectasis, which occurred in 8 patients (10.00%) and 5 patients (6.25%) in the IPREs group and in 18 patients (22.50%) and 13 patients (16.25%) in the control group, respectively. No significant differences were observed in the incidence of other PPCs between the two groups(*P >*0.05; **Figure 3**). Subgroup analysis stratified by surgical approach indicated that among patients undergoing open surgery, the IPREs group had a significantly lower incidence of PPCs (*P* = 0.005). Furthermore, analysis based on surgical procedures demonstrated that, compared with the control group, patients in the IPREs group who underwent hepatectomy and pancreatectomy had a lower incidence of PPCs, with P values of 0.016 and 0.002, respectively (**Table 5**).

**Table 5:** Results of subgroup analysis by surgical method.

Surgical methods	Total	PPCs event in IPREs group (n=80)	PPCs event in control group (n=80)	*P*	OR(95%CI)
Surgical approaches					
Open surgery	41/119	13/59	28/60	0.005	0.323(0.145-0.717)
Laparoscopic surgery	8/41	5/21	3/20	0.697^1)^	1.771(0.363-8.648)
Surgical procedures					
**Hepatectomy**	15/72	4/39	11/33	0.016	0.229(0.065-0.808)
left hemihepatectomy	6/20	2/11	4/9	0.336^1)^	0.278(0.037-2.092)
right hemihepatectomy	4/18	1/8	3/10	0.588^1)^	0.333(0.028-4.036)
caudate lobectomy	2/20	0/12	2/8	0.147^1)^	1.133(0.894-1.989)
two combined segmentectomies	3/14	1/8	2/6	0.538^1)^	0.286(0.019-4.237)
**Biliary procedure**	14/49	8/22	6/27	0.276	2.000(0.570-7.023)
radical surgery for cholangiocarcinoma	5/16	4/9	1/7	0.308^1)^	4.800(0.397-58.013)
radical cholecystectomy for gallbladder cancer	5/25	2/8	3/17	1.000^1)^	1.556(0.205-11.829)
cholangioenterostomy	4/8	2/5	2/3	1.000^1)^	0.333(0.017-6.654)
**Pancreatectomy**	20/39	5/19	15/20	0.002	0.119(0.028-0.501)
distal pancreatectomy	10/23	3/12	7/11	0.100^1)^	0.190(0.032-1.145)
pancreaticoduodenectomy	10/16	2/7	8/9	0.035^1)^	0.050(0.004-0.706)

IPREs, intensive pulmonary rehabilitation exercises; PPCs, postoperative pulmonary complications; OR, odds ratio. The names of the three surgical procedures (hepatectomy, biliary procedure, and pancreatectomy) are bolded to emphasize the comparison of total postoperative pulmonary complications (PPCs) between the two groups.

1) Fisher’s exact probability.

### 3.5 Other major surgical complications and hospital utilization

**Figure 4:**
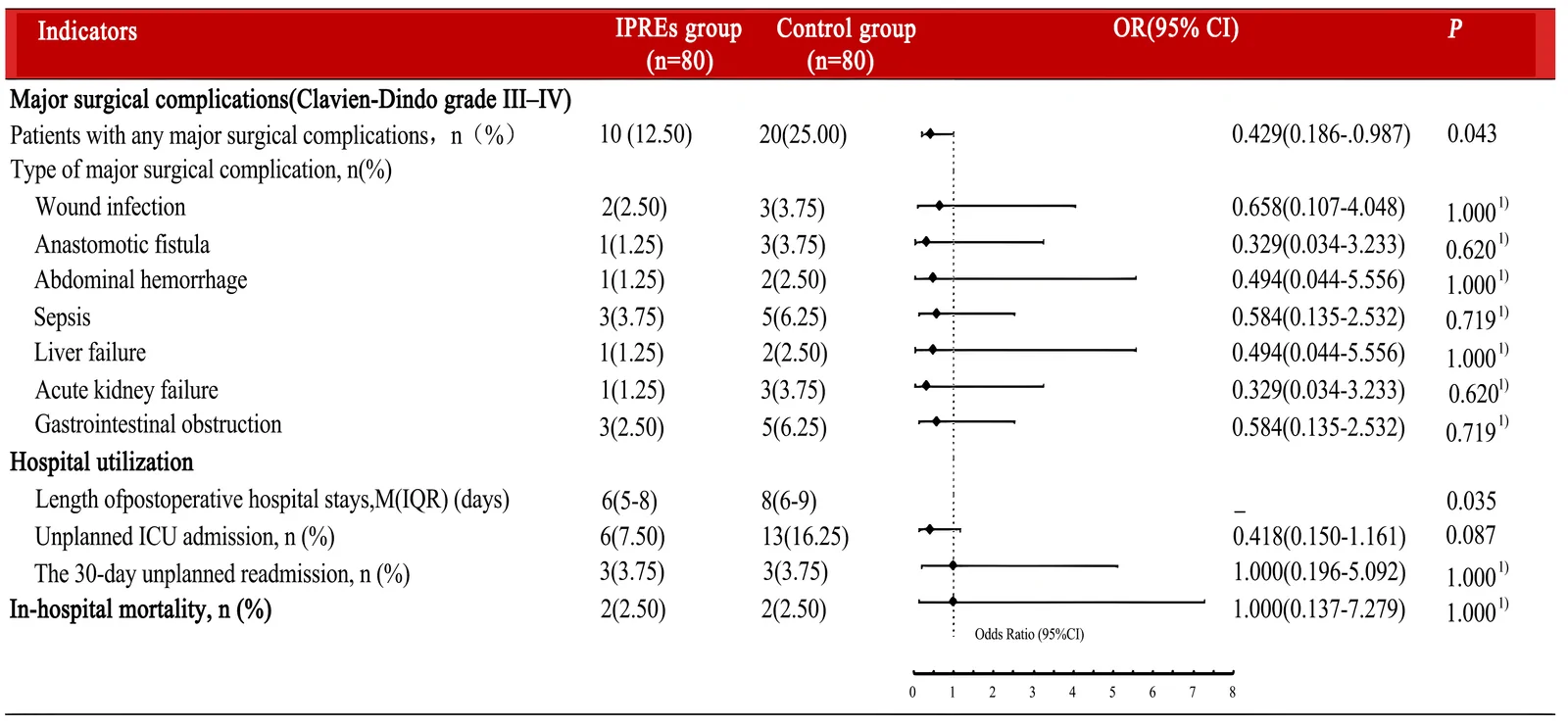
Comparison of major surgical complications and key hospital utilization metrics between groups. IPREs, intensive pulmonary rehabilitation exercises; OR, odds ratio. ICU, intensive care unit; M(IQR): median (interquartile range); 1) Fisher’s exact probability.

Regarding major surgical complications (Clavien-Dindo grade ≥ III) within the first 30 postoperative days, the incidence was significantly lower in the IPREs group than in the control group (12.50% vs. 25.00%; OR: 0.429; 95% CI, 0.186–0.987; *P* = 0.043). But no significant differences were observed between the groups in the rates of any specific complications (all *P >*0.05; **Figure 4**). Furthermore, for hospital utilization, the postoperative hospitalization duration was longer in the control group than in the IPREs group (6 days [IQR, 5-8] vs. 8 days [IQR, 6–9 vs., *P* = 0.035). Unplanned ICU admission rates were comparable between the two groups [6 (7.5%) vs. 13 (16.25%), OR: 0.418; 95% CI: 0.150–1.161; *P* = 0.087]. In the IPREs group, 4 patients required unplanned ICU admission because of PPCs, and 2 patients required unplanned ICU admission because of abdominal hemorrhage. In the control group, 11 patients had unplanned ICU admissions due to PPCs, while the other two admissions were attributed to acute kidney failure and abdominal hemorrhage, respectively. The rates of 30-day unplanned readmission and in-hospital mortality were also similar between the two groups (*P >*0.05; **Figure 4**).

## 4 Discussion

Effective prevention of PPCs is critical for improving recovery outcomes in patients undergoing major abdominal surgery. This clinical trial evaluated the efficacy of a nurse-led, intensive short-term perioperative pulmonary rehabilitation program in reducing PPCs among frail elderly patients undergoing HBP surgery. Compared with conventional self-directed respiratory training, the IPREs program significantly improved adherence to the “I Cough” protocol, reduced PPC incidence, and shortened postoperative hospital stay.

Although all patients received standardized respiratory rehabilitation instructions upon admission, significant between-group differences emerged in adherence to the “I Cough” protocol. The control group demonstrated poor overall compliance (approximately 33.3%), with the lowest adherence rates (20%–27.5%) observed for key components including ambulation, incentive spirometry, and coughing/deep breathing exercises. These findings indicate substantial challenges in self-managing perioperative bundled rehabilitation protocols among frail elderly individuals and are consistent with those reported by Cassidy et al. (12). In contrast, the IPREs group achieved markedly higher adherence rates (67.5% to 82.5% across components), representing an approximate 50% overall improvement. The consistent involvement of rehabilitation nurses in daily program planning and implementation was instrumental in driving this enhanced adherence through professional guidance, direct communication, and psychological support. While family assistance was available to control group patients, the absence of professional engagement resulted in suboptimal participation. Furthermore, nurse-led standardization of pulmonary rehabilitation protocols established clear outcome expectations, bolstering patients’ confidence in achieving rehabilitation goals.

Regarding functional recovery, both groups exhibited postoperative declines in inspiratory volume and handgrip strength; however, these reductions were less pronounced in the IPREs group. By postoperative day (POD) 5, inspiratory volume and handgrip strength in the intervention group had returned to preoperative baseline levels, whereas the control group continued to show significant deficits. These results suggest that the nurse-led intervention facilitated more effective pulmonary and muscular recovery. Additionally, the IPREs group reported significantly lower pain scores at rest and during mobilization on POD 3 and 5, with pain intensity predominantly mild to moderate. In contrast, the control group reported moderate to severe pain during the same period. This difference may be attributed to rehabilitation nurses’ role in enhancing patient motivation, engagement, and confidence, thereby fostering greater commitment to the pulmonary improvement program. These psychological and behavioral benefits likely contributed to reduced pain perception and decreased analgesic use. Moreover, time to independent ambulation was significantly shorter in the intervention group (42.31 ± 9.56 hours) compared with controls (78.22 ± 10.21 hours; *P* < 0.001). Although achieving independent walking at approximately 42 hours may not be considered “early” relative to younger surgical populations, it represents a clinically meaningful improvement for frail elderly patients undergoing major HBP surgery. This improvement can be largely explained by the consistent face to face guidance and behavioral coaching provided by rehabilitation nurses, which enhanced patient confidence and adherence to mobilization regimens, helping overcome barriers to early postoperative activity.

The IPREs group demonstrated a significantly lower overall incidence of PPCs, with notably reduced rates of pneumonia and atelectasis (*P* < 0.05). This clinical benefit is primarily attributable to the implementation of a nurse-led, comprehensive perioperative pulmonary rehabilitation program that facilitated standardized application of the “I Cough” protocol. Through twice-daily face to face guidance, rehabilitation nurses promptly identified and addressed patient specific barriers to protocol adherence, effectively enhancing compliance with core strategies including respiratory exercise, oral care, head of bed elevation, early ambulation, and handgrip strength training. Evidence suggests these standardized interventions facilitate alveolar recruitment, maintain functional residual capacity, and improve airway clearance (12, 23), collectively promoting postoperative pulmonary recovery. Additionally, early mobilization and respiratory training may reduce pulmonary infection risk by modulating immune and inflammatory responses (24, 25). However, this study cohort comprises various HBP surgical procedures, including hepatectomy, biliary surgery, and pancreatic resections, which differ substantially in operative trauma, postoperative complication profiles, and baseline risk. For instance, pancreatic resections typically involve more extensive lymphadenectomy and gastrointestinal reconstruction, leading to different postoperative pathways compared with hepatic resections. To investigate the differential effects of the pulmonary rehabilitation program across surgical types, we conducted subgroup analyses. The results indicated that the IPREs program conferred greater benefit in patients undergoing open, hepatic, or pancreatic surgery, whereas those receiving laparoscopic or biliary procedures showed more modest improvements. This differential effect likely arises from underlying physiological mechanisms. Open HBP resections, particularly major pancreatectomies and hepatectomies, often involve extensive bilateral subcostal incisions associated with significant postoperative pain. Such pain can exacerbate pre-existing diaphragmatic dysfunction and impaired respiratory mechanics in frail elderly patients (4, 11), which represent key pathophysiological targets of intensive respiratory rehabilitation. In contrast, laparoscopic and minimally invasive biliary procedures generally cause milder postoperative pain with less impact on diaphragmatic function, potentially explaining the attenuated benefits observed in these subgroups. Given the small sample sizes in the subgroups, the subgroup analyses by surgical approach (laparoscopic vs. open) and procedure type (hepatectomy, biliary surgery, pancreatic resection) should be interpreted with caution. These analyses are exploratory and hypothesis generating only. They are intended to inform the design of future, adequately powered studies and should not be considered confirmatory.

The present study demonstrated that patients receiving the IPREs intervention exhibited a significantly lower overall incidence of major postoperative complications. No adverse events, including falls, hemorrhage, or wound dehiscence, were reported in either group, supporting the safety and efficacy of the nurse-led “I Cough” program in frail elderly patients undergoing HBP surgery (26, 27). Furthermore, the intervention group experienced a significantly shorter postoperative hospital stay (mean reduction: 2 days, *P* < 0.05) and showed a trend toward a lower unplanned ICU admission rate (6/80 vs. 13/80), although this difference did not reach statistical significance (*P* = 0.087). Further analysis of ICU-admitted patients revealed that only 2 patients (33.3%) in the IPREs group were admitted due to PPCs, compared with 11 patients (84.6%) in the control group. Although not statistically significant, this difference carries notable clinical relevance, given that PPCs are a leading cause of unplanned ICU transfers, endotracheal intubation, and prolonged hospitalization in frail elderly surgical populations. Notably, the reduction in Clavien-Dindo grade ≥III complications was not attributable to any single complication type. Instead, the improvement in this composite endpoint reflects a multifactorial benefit. Overall PPCs, particularly pneumonia and atelectasis, were significantly reduced. Enhanced pulmonary function and early mobilization may have indirectly lowered the risk of other extrapulmonary complications, such as gastrointestinal dysfunction, cardiovascular events, and infectious complications, although the present study did not demonstrate statistical significance in comparisons of individual complications. In addition, the perioperative pulmonary rehabilitation program also exerted a broader positive impact on postoperative recovery, including a decrease in unplanned ICU admissions and prolonged hospital stays secondary to severe pulmonary events. Collectively, these benefits are best understood as an integrated effect of the pulmonary rehabilitation strategy on the overall postoperative recovery trajectory. Importantly, the introduction of dedicated rehabilitation nurses did not result in a significant increase in nursing labor costs. These nurses played a pivotal role in the implementation of the ERAS protocol, particularly with respect to pulmonary rehabilitation, and helped ensure consistent adherence to standardized rehabilitation plans among high-risk frail elderly patients. Taken together, these efforts contributed to a reduction in major postoperative complications, improved patient satisfaction, and a decrease in overall healthcare expenditures.

From an oncological perspective, uninterrupted or timely adjuvant therapy is paramount for achieving optimal outcomes. The significant reduction in PPCs and consequent shortening of hospital stay observed in this study represent more than perioperative quality metrics. They may serve as critical facilitators for faster “Return to Intended Oncologic Therapy” (RIOT) (28). By mitigating major postoperative setbacks in frail elderly patients, this protocol could help minimize delays in initiating or resuming essential chemotherapy or radiotherapy, thereby preserving intended treatment trajectories and therapeutic efficacy. Future studies with longer follow-up are needed to directly evaluate the impact of such enhanced recovery pathways on RIOT rates and long-term survival. Furthermore, it is important to recognize that the “pancreatic surgery” subgroup in this study actually encompasses several distinct cancer entities, including pancreatic ductal adenocarcinoma, distal cholangiocarcinoma, and ampullary carcinoma. As highlighted by Uijterwijk et al. (29), these periampullary cancers are biologically and prognostically distinct, rather than a single uniform category. This heterogeneity may influence patients’ baseline physiological reserve and their response to pulmonary rehabilitation. Future subgroup analyses should consider stratifying by tumor type to explore these potential differences. Meanwhile, a substantial proportion of patients in our cohort underwent hepatectomy. The postoperative risk in hepatobiliary surgery is known to be highly individualized, influenced by factors such as liver parenchymal quality (e.g., steatosis, fibrosis, cirrhosis), extent of resection, surgical complexity (e.g., as in perihilar cholangiocarcinoma), and patient comorbidities. As demonstrated by Golse et al. (30), personalized preoperative nomograms can effectively predict postoperative risks in complex hepatobiliary resections. Therefore, while our pulmonary rehabilitation program demonstrated overall benefit, future efforts to optimize recovery in hepatectomy patients should incorporate individualized risk stratification to tailor the intervention intensity.

## 5 Conclusion

This clinical trial demonstrates that for the vulnerable population of frail elderly patients undergoing HBP surgery, implementing a nurse-led intensive respiratory rehabilitation program can significantly improve adherence to the “I Cough” protocol, reduce the incidence of PPCs, shorten hospital stays, and enhance postoperative recovery outcomes.

## 6 Limitations

This study has several limitations. First, as a single-center study, this research did not fully incorporate individualized factors that may influence the effectiveness of pulmonary rehabilitation exercises, including patient disease characteristics, comorbidities, and surgical trauma. Second, frailty was assessed solely using the FRAIL scale without complementary objective measures, which may introduce measurement bias. Third, the non-blinded, high contact intervention poses a risk of performance bias, particularly for subjective outcomes such as self-reported pain (VAS score). Future multicenter studies that incorporate individualized risk stratification and more objective assessment tools are warranted to validate the long-term benefits of the IPREs protocol in frail elderly patients undergoing hepatobiliary and pancreatic HBP surgery.

## Data Availability

The raw data supporting the conclusions of this article will be made available by the authors, without undue reservation.
